# Has the COVID-19 Pandemic Increased Advance Care Planning Discussions Held by Older Adults?

**DOI:** 10.1093/geroni/igab046.512

**Published:** 2021-12-17

**Authors:** Gloria Gutman, Brian deVries, Robert Beringer, Paneet Gill, Helena Daudt

**Affiliations:** 1 Simon Fraser University, Vancouver, British Columbia, Canada; 2 San Francisco State University, San Francisco, California, United States; 3 University of Victoria, Victoria, British Columbia, Canada; 4 Simon Fraser University, Surrey, British Columbia, Canada; 5 Victoria Hospice, Victoria, British Columbia, Canada

## Abstract

In an online survey exploring older Canadians’ experiences during the COVID-19 pandemic, 3989 respondents aged 55-99 indicated whether they had discussed their future care preferences and with whom, prior to and since the outbreak. Pre-pandemic, 62% had held such discussions; since the pandemic 43% did so, 11% for the first time. Rates were significantly higher among white respondents than among persons of color, women than men, and those 65+ than younger respondents. Respondents were most likely to have talked, respectively, with their spouse (58% before; 40% since), family (35%; 22%), and friends (12%; 10%)—with higher rates for white, women and older respondents. Surprisingly, only 4% before and 2% since the pandemic had discussed their care preferences with a doctor. Initiation of some new discussions was encouraging but there were fewer than expected, perhaps due to denial, superstition, or disbelief of pandemic severity. Advance care planning remains an under-utilized resource.

